# Multiple subcutaneous tuberculous abscesses in a dermatomyositis patient without pulmonary tuberculosis: a case report and literature review

**DOI:** 10.1186/s12879-020-05137-w

**Published:** 2020-06-12

**Authors:** Weiwei Gao, Yi Zeng, Wei Chen

**Affiliations:** 1 Department of Tuberculosis, the second hospital of Nanjing, Nanjing University of Chinese Medicine, 1-1 Zhongfu Road, Gulou district, Nanjing, 210003 Jiangsu province China; 2 Clinical Research Center, the second hospital of Nanjing, Nanjing University of Chinese Medicine, 1-1 Zhongfu Road, Gulou district, Nanjing, 210003 Jiangsu province China

**Keywords:** Tuberculous, Abscess, Subcutaneous, Limb, Dermatomyositis

## Abstract

**Background:**

Even though remarkable progress for diagnostics of pulmonary TB has been made, it is still a challenge to establish a definitive diagnosis for extrapulmonary TB (EPTB) in clinical practice. Among all the presentations of EPTB, cold abscesses are unusual and deceptive, which are often reported in the chest wall and spine. Subcutaneous abscess in the connective tissue of limbs is extremely rare.

**Case presentation:**

A 48-year-old man with dermatomyositis was hospitalized because of multiple subcutaneous tuberculous abscesses in his limbs, but without pulmonary tuberculosis. Particularly, one insidious abscess appeared during anti-TB treatment due to “paradoxical reaction”. After routine anti-TB therapy, local puncture drainage and surgical resection, the patient was cured and discharged.

**Conclusions:**

Tuberculous infection should be kept in mind for the subcutaneous abscess of immunocompromised patients, even without previous TB history. Treatment strategy depends on the suppurating progress of abscess lesions. Meanwhile, complication of newly-developed insidious abscess during treatment should be vigilant.

## Background

Tuberculosis (TB) is still a major issue for public health in the developing countries even through tremendous efforts [1]. Its causative agent, *Mycobacterium tuberculosis* (MTB) mainly invades the lungs and causes pulmonary TB (PTB). Recently, the occurrence rate of extrapulmonary TB (EPTB) presents a significant rising trend, especially among people with immunocompromise [2, 3], and accounts for 15–30% of all the TB cases [4]. EPTB could originate from either endogenous or exogenous infection. The tricky point is, it is hard to establish a definitive diagnosis for EPTB, and prone to delay treatment, since the clinical symptoms and imaging characteristics are usually diverse and vague [5]. Among all the presentations of EPTB, cold abscesses are unusual and deceptive [6, 7]. Tuberculous abscess is often observed in the chest wall and spine [8–10]. Subcutaneous tuberculous abscess refers to MTB infection in the subcutaneous connective tissue and skeletal muscle, which is an extremely rare type of EPTB [11]. Only 5 cases are reported in limbs in PubMed database from 2000 to 2019. In this report, we presented a middle-aged man with dermatomyositis who suffered from multiple subcutaneous tuberculous abscesses in his limbs, but without PTB.

## Case presentation

A 48-year-old man was admitted to our hospital because of tuberculous abscesses. The patient had been diagnosed as dermatomyositis in another hospital since one year ago and had taken low dose prednisolone (15 mg/d) continuously. One month before hospitalization, he unconsciously noticed two swellings in his limbs without pain and redness. The patient was suspected of TB infection in another hospital and transferred to our hospital, which is the designated hospital for infectious diseases in Nanjing district. At admission, the patient had no other symptoms, such as tenderness, redness, fever, cough or night sweats. In addition, his medical history showed that he had neither underlying disease, like diabetes, hypertension, or coronary heart disease, nor trauma and intramuscular injection recently. Neither he nor his family had previous history of TB ever.

**Fig. 1:**
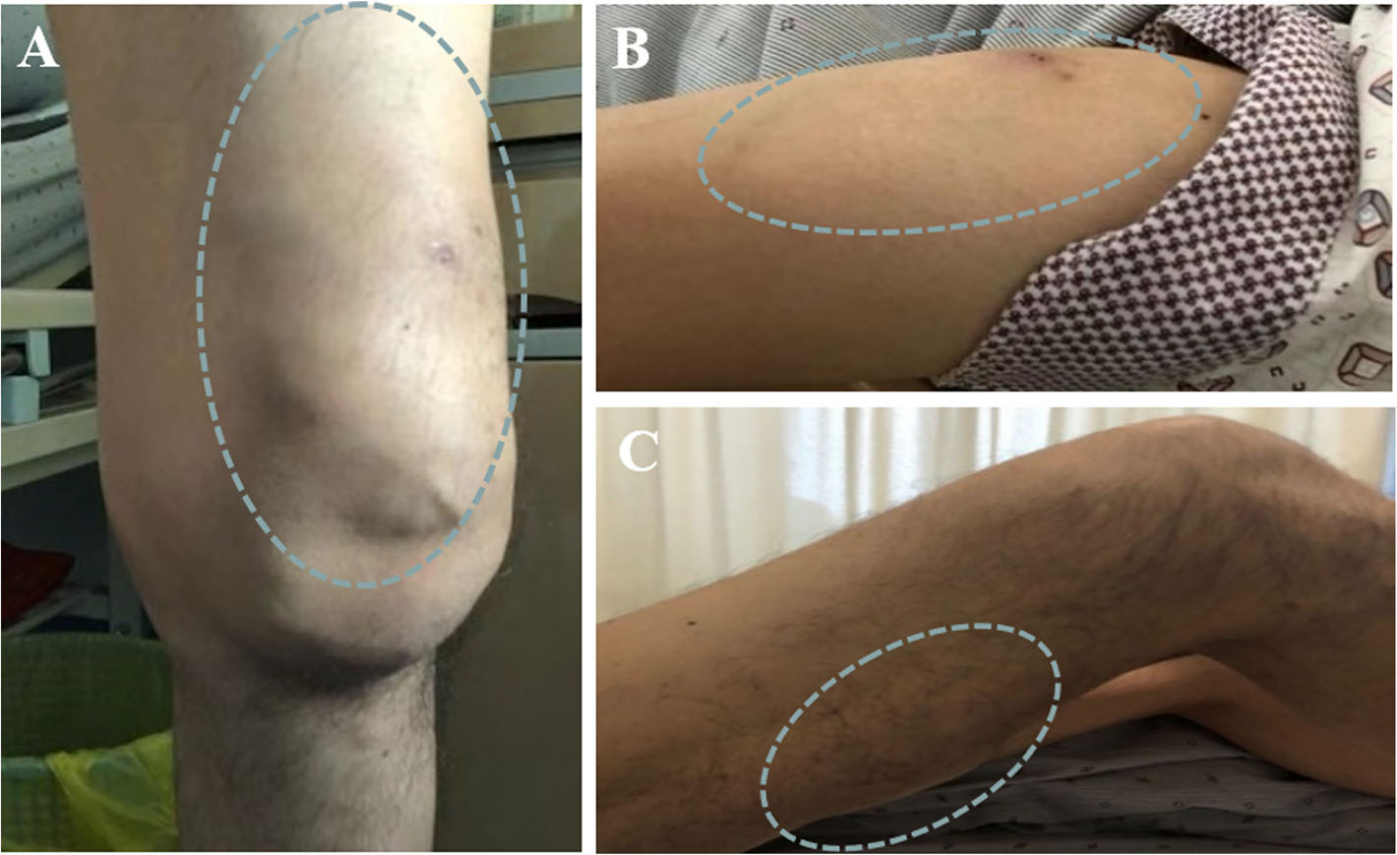
Localization of three swellings in the limbs. One soft-tissue swelling on the tront of left femur (ca. 5.0 × 12.0 cm) (A), one soft-tissue swelling on the left lower humeru (ca. 4.0 × 5.0 cm) (B), and another mass on the right femur above the right armpit (ca.6.0 × 8.0 cm) (C). The sites of abscesses were defined by circles

**Fig. 2:**
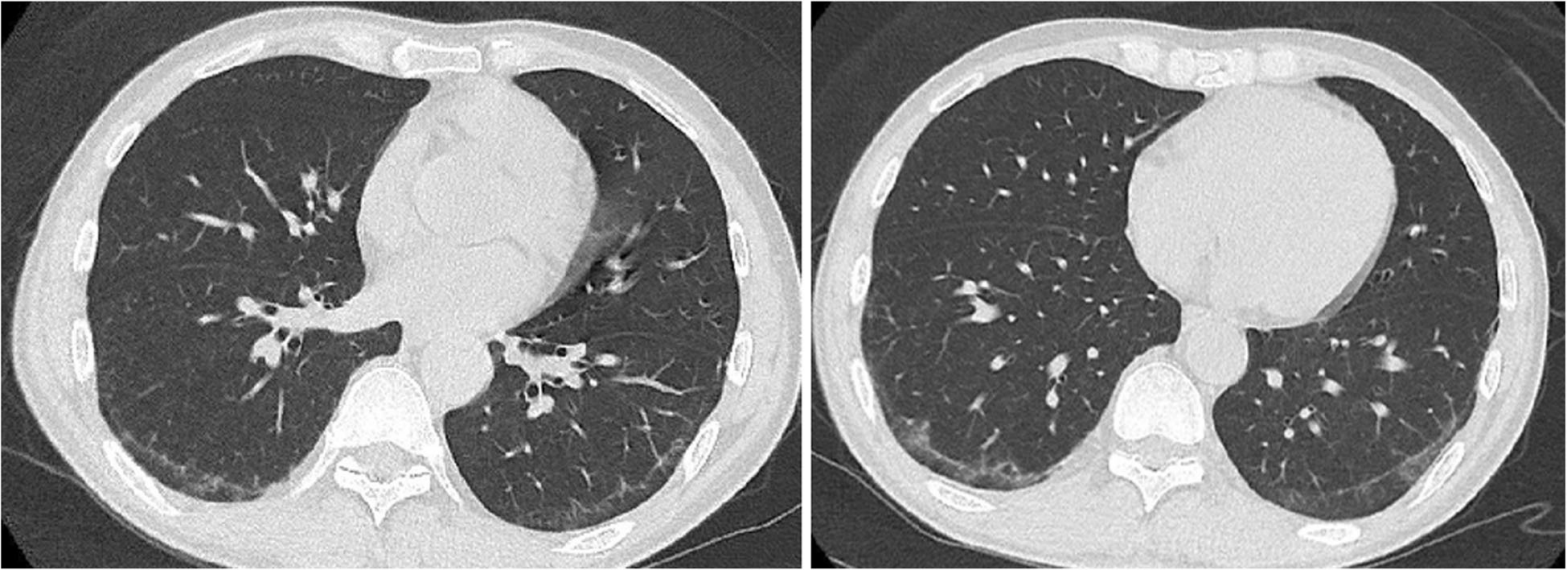
Chest CT scan showed interstitial change in both lower lungs under the pleur without active TB lesion

**Fig. 3:**
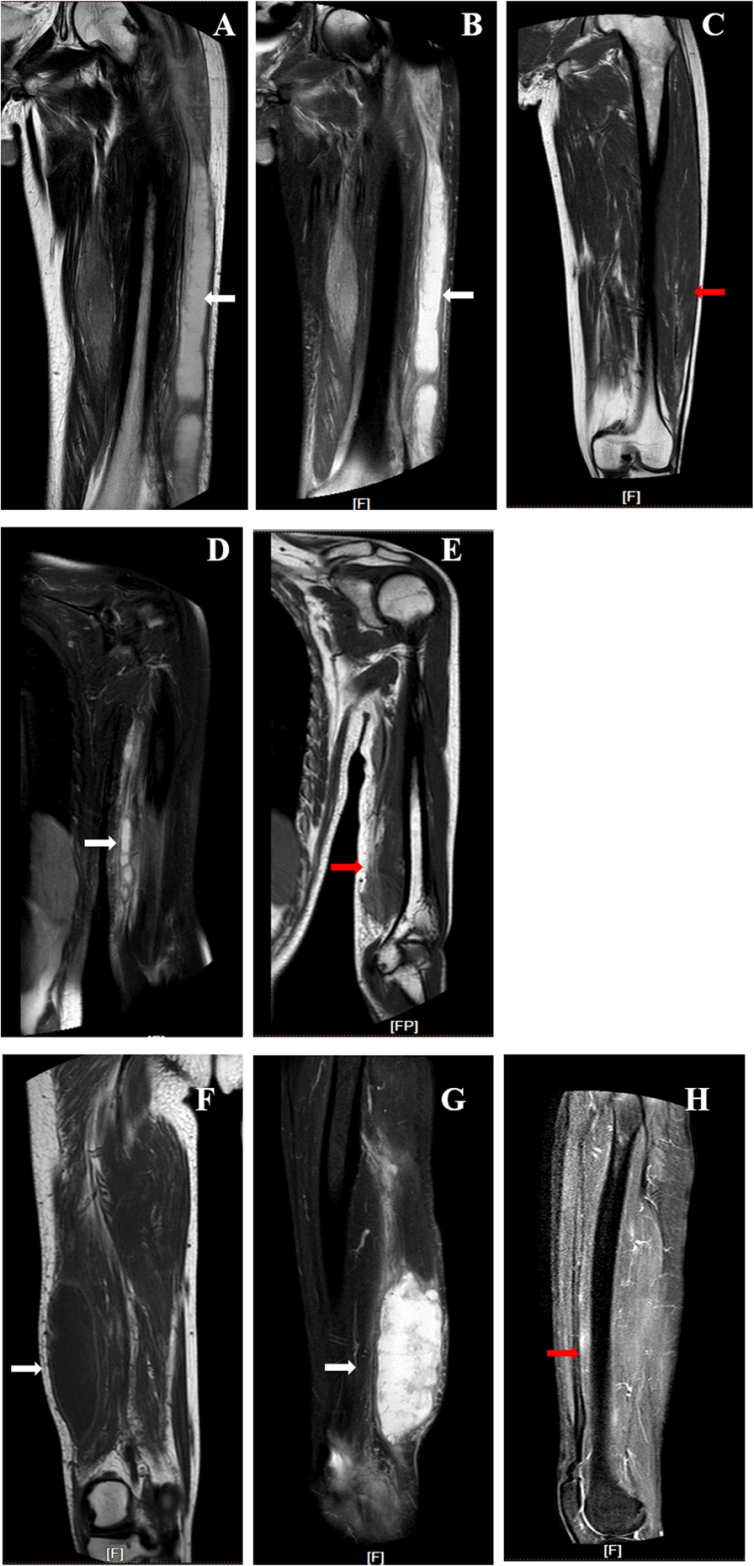
MRI of subcutaneous abscesses in the limbs pre and post treatment. MRI of the left femoral showed two different fluid collections extended along the path of subcutaneous connective tissue (upper panel, A and B). There was a spot with slightly high signal at the lower end of the left humerus (middle panel, D). After the comprehensive treatment, the left femur and the left humerus abscesses faded away obviously (C and E). Another mass was on the right femur above the right popliteal fossa, without fluid fluctuation (lower panel, F and G). After surgical removal of the abscesses, the abscess lesion on the right humerus was restored (H)

Physical examinations revealed two soft tissue swellings on the left lower humeru and the tront of left femur, approximately 4.0 × 5.0 cm and 5.0 × 12.0 cm, respectively. The overlying skins presented with normal temperature, scars, rash or sinuses (Fig. 1A and B). A systematic laboratory examination of the patient did not find any abnormities for blood routine test, liver and renal function tests, common neoplasms, the cardiovascular and neurological functions. The level of NT-proBNP, neoplastic markers, anti-neutrophil cytoplasmic antibodies, C3, C4 and IgG4 were negative or normal. C-reactive protein was 12.9 mg/dL, and the erythrocyte sedimentation rate value was 80 mm/h. Computed tomography (CT) scans did not find any active TB lesion in the lung (Fig. 2). Magnetic resonance imaging (MRI) of the left humerus and the left femoral showed two different fluid collection extension along the path of subcutaneous connective tissue. The abscess on the left femoral penetrated the posterior abdominal wall musculature and formed a sinus tract (Fig. 3).

The pus isolated from the subcutaneous abscesses by needle aspiration was positive for Ziehl-Neelsen staining (2+). qPCR confirmed the presence of MTB DNA. GeneXpert MTB/RIF assay showed that the MTB strain was susceptible to rifampicin. Drug susceptibility test further revealed that the cultured MTB strain was susceptible to all the first-line anti-TB drugs. Once upon confirming the tuberculous lesions, anti-TB regimen was initiated, including isoniazid (600 mg qd), rifampicin (600 mg qd), ethambutol (750 mg qd), pyrazinamide (1000 mg qd). Meanwhile, pus drainage was conducted once every other day. 50 ml and 200 ml of yellow pus were extracted per puncture from the abscess of the left humerus and the left femoral, respectively, followed by direct injection of 0.5 g and 1.0 g streptomycin. No complications occurred during the process of puncture, drainage and drug injection. After two months of treatment, the two abscesses faded obviously (Fig. 3).

Unfortunately, another subcutaneous lump emerged at the back of the right thigh, 6.0 × 8.0 cm in size, without warm, redness or pain (Fig. 1C). Different from the previous two abscesses, there was no fluid fluctuation within this lump when touched. A needle aspiration only got little pus, which was remarkably MTB-positive. Since the hard lump did not suppurate, it was excised by surgical operation. Histopathological examinations of the lump tissue revealed caseous necrosis and tuberculous granulomas inflammation. One week after surgery, the patient with wound healing well was discharged and accepted postoperative anti-TB medication for 12 months. At one-year follow-up, MRI did not find any lump or abscess recurrence.

## Discussion and conclusion

**Fig. 4:**
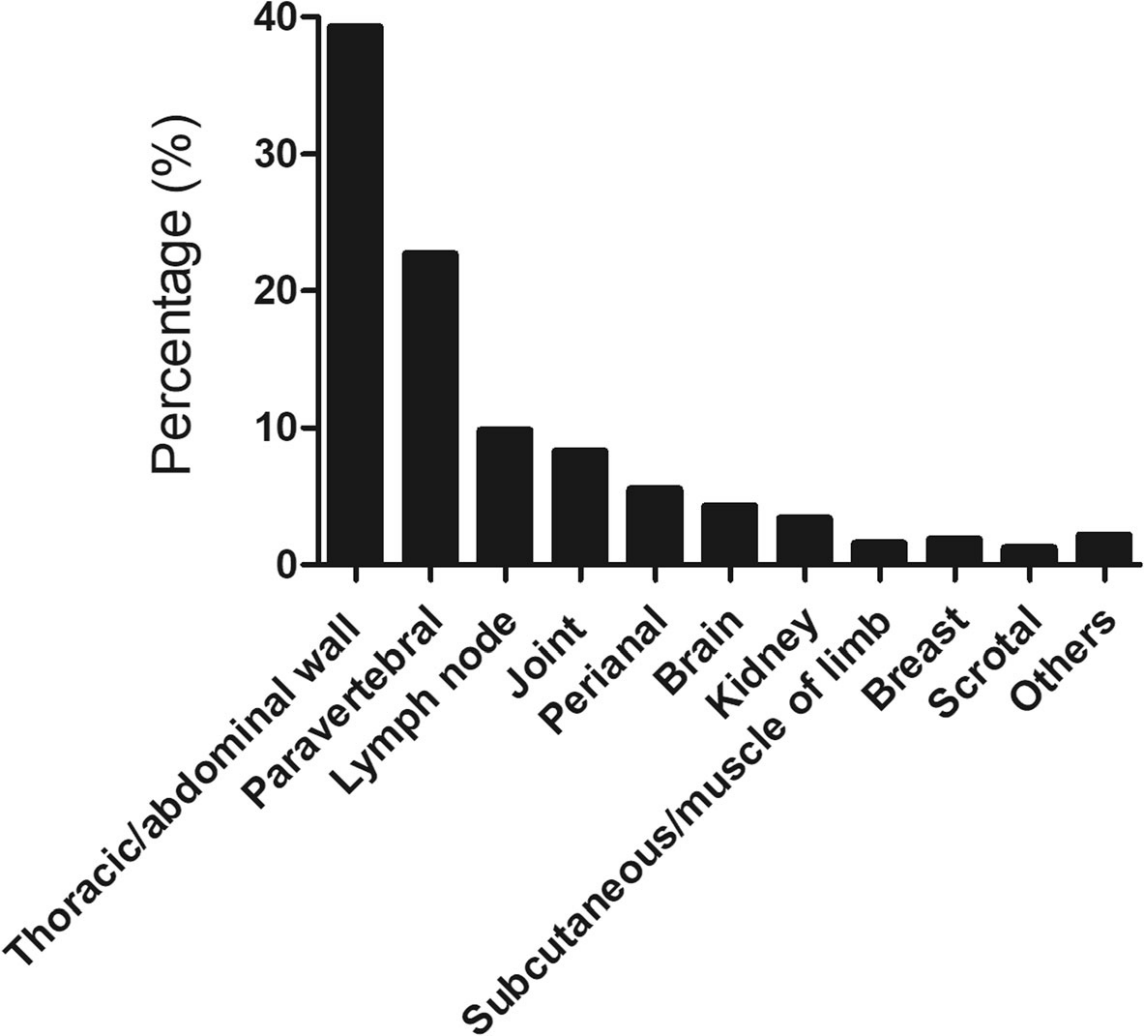
Distribution of different TB abscess lesions from 2000 to 2019

We performed a retrospective study of patients with tuberculous abscess in the PubMed database, with keywords “tuberculosis” and “abscess”. Only the articles in English were counted. From 2000 to 2019, 329 cases of tuberculous abscess are reported totally. The predominant site of tuberculous abscess is the thoracic and abdominal wall (39.2%), followed by paravertebral line (22.70%) and lymph node (9.81%) (Fig. 4). In these cases, there are only 5 cases (1.53%) occurring in the subcutaneous and muscle tissues of limbs, 2 males and 3 females, with average age of 50.2. Their clinical presentations are varied and non-specific, including chronic swelling with pain and cough or sputum. All of these patients have active PTB. 3 patients have underlying diseases, such as renal allograft and rheumatoid arthritis. Multiple big abscesses are observed for all of them and the most frequent sites are the thigh. The 2 patients without underlying diseases had single and small lesion. We noticed that the most frequent sites are the thigh. Luckily, like our patient, all the 5 patients have very good prognosis after treatment (summarized in Table S1).

How are the subcutaneous tuberculous abscesses are caused? One of the high-risk factors is PTB, where the lung might be the primary infection site. MTB disseminated from the lung remains latent in the subcutaneous tissues until adverse conditions like immunosuppression or malnutrition [12]. However, in our case, the patient had no active or previous PTB, which excluded the possibility of pulmonary dissemination. On the other side, trauma, injury or local injection might cause subcutaneous and muscle tuberculous infection through direct introduction of mycobacteria [13]. In our case, the patient recently had neither trauma nor injection. It’s worthy to note that the patient had dermatomyositis, with long-term administration of hormones. He was at an immunocompromised condition and susceptible to variable infections. In addition, dermatomyositis mainly impairs the skin and muscle of the limb [14, 15]. Once infection, pathogens are difficult to be eliminated and prone to spread along the fascia and muscular tissues [16]. Therefore, we inferred that dermatomyositis promoted the development of subcutaneous tuberculous abscesses.

As for the third abscess at the back of the right thigh, it was very insidious and not perceived at the first physical examination. This abscess appeared after two-month anti-TB therapy, when another two foci showed obvious absorption. This was reminiscent of a similar case where a woman with miliary TB developed subcutaneous abscesses after 5 months of anti-TB treatment [17]. Actually, the phenomenon is defined as “paradoxical reaction”, which manifests as deterioration of pre-existing tuberculous lesions or development of new lesions during anti-TB treatment [18, 19]. Paradoxical tuberculous reaction is more common in the HIV-positive TB patients [20].

The management of TB subcutaneous tuberculous abscess includes pharmacological and surgical treatment, with drainage and surgical debridement. The challenge to treat tuberculous abscess only lies in anti-TB drugs, since it is difficult for them to penetrate the wall of a pyogenic abscess, leading to an unsatisfactory outcome. Pus puncture followed by drug injection enhances the efficacy of anti-TB therapy, reduces surgical trauma, and avoids postoperative complications, making it a safe and feasible treatment option for tuberculous abscesses [21, 22]. It’s reported that surgical treatment performed during the exudative phase of lumbar spine TB significantly increased the incidence of abscess recurrence, unhealed lesions, and sinus tract formation [23]. Hence, preoperative anti-TB therapy is necessary for patients with tuberculous abscesses. On the other hand, treatment strategy often depends on the progress of the abscess lesion. If the lesion is suppurated, puncture and local injection of anti-TB drugs are recommended. If the lesion is not suppurated or could not be extracted by puncture, surgical resection should be considered.

In conclusion, we described an extremely rare case about a dermatomyositis patient with multiple subcutaneous tuberculous abscesses in the limbs, but without PTB. Through pus puncture, surgical resection, and comprehensive anti-TB regimen, the patient was successfully cured and discharged. Tuberculous infection should be kept in mind when an unexplained swelling occurred in the limbs of immunocompromised patients, even though no previous TB history.

## Supplementary information

**Additional file 1 Table 1** Clinical characteristics of patients with tuberculous abscess in limbs.

**Additional file 2.**

## Data Availability

Data relating to this study are contained and presented in this document. Other materials are available from the corresponding authors on reasonable request.

## Abbreviations

TB: Tuberculosis
PTB: Pulmonary tuberculosis
EPTB: Extrapulmonary tuberculosis
MTB: *Mycobacterium tuberculosis*
MRI: Magnetic resonance imaging
CT: Computed tomography

## References

[1] Global tuberculosis report 2019, Geneva: World Health Organization. 2019, Licence: CC BY-NC-SA 3.0 IGO.

[2] Gomes da Rocha Dias AP, von Amann B, Costeira J, Gomes C, Bárbara C: Extrapulmonary tuberculosis in HIV infected patients admitted to the hospital. European Respiratory Journal 2016, 48(suppl 60):PA2761.

[3] Sester M (2014). Leth Fv, Bruchfeld J, Bumbacea D, Cirillo DM, Dilektasli AG, Domínguez J, Duarte R, Ernst M, Eyuboglu FO et al: risk assessment of tuberculosis in Immunocompromised patients. A TBNET study. Am J Respir Crit Care Med.

[4] Rodriguez-Takeuchi SY, Renjifo ME, Medina FJ (2019). Extrapulmonary tuberculosis: pathophysiology and imaging findings. RadioGraphics.

[5] Ramirez-Lapausa M, Menendez-Saldana A, Noguerado-Asensio A (2015). Extrapulmonary tuberculosis: an overview. Rev Esp Sanid Penit.

[6] Brown S, Thekkinkattil DK (2016). Tuberculous cold abscess of breast: an unusual presentation in a male patient. Gland Surg.

[7] Dv K, Gunasekaran K, Mishra AK, Iyyadurai R: Disseminated tuberculosis presenting as cold abscess of the thyroid gland—a case report. Oxford Medical Case Reports 2017, 2017(9). 10.1093/omcr/omx049 PMC5597852 28928976

[8] Hussain S (2016). Chest wall tuberculous ulcer: a rare complication of pulmonary tuberculosis. Indian J Tuberc.

[9] Hsu H-E, Chen C-Y: Tuberculous retropharyngeal abscess with Pott disease and tuberculous abscess of the chest wall: A case report. Medicine 2019, 98(27). 10.1097/MD.0000000000016280 PMC6635237 31277156

[10] Srivastava AK, Sardhara J, Godbole C, Behari S: Surgical Treatment of Spinal Tuberculosis Complicated with Extensive Abscess. In: Tuberculosis of the Central Nervous System. edn.: Springer; 2017: 447–459.

[11] Sezgin B, Atilganoglu U, Yigit O, Ergün SS, Cambaz N, Demirkesen C (2008). Concomitant cutaneous metastatic tuberculous abscesses and multifocal skeletal tuberculosis. Indian J Dermatol.

[12] Pacheco C, Silva E, Miranda J, Duarte R (2015). Cutaneous tuberculosis as metastatic tuberculous abscess. J Bras Pneumol.

[13] Kothavade R, Dhurat R, Mishra S, Kothavade U (2013). Clinical and laboratory aspects of the diagnosis and management of cutaneous and subcutaneous infections caused by rapidly growing mycobacteria. Eur J Clin Microbiol Infect Dis.

[14] Hayeri MR, Ziai P, Shehata ML, Teytelboym OM, Huang BK (2016). Soft-tissue infections and their imaging mimics: from cellulitis to necrotizing fasciitis. RadioGraphics.

[15] Malik A, Hayat G, Kalia JS, Guzman MA (2016). Idiopathic inflammatory myopathies: clinical approach and management. Front Neurol.

[16] Chen I-J, Tsai W-P, Wu Y-JJ, Luo S-F, Ho H-H, Liou L-B, Chen J-Y, Kuo C-F, Chang H-C, Yang C-H (2010). Infections in polymyositis and dermatomyositis: analysis of 192 cases. Rheumatology.

[17] Mert A, Bilir M, Ozturk R, Tabak F, Ozaras R, Tahan V, Senturk H, Aktuglu Y (2000). Tuberculous subcutaneous abscesses developing during miliary tuberculosis therapy. Scand J Infect Dis.

[18] Brown CS, Smith CJ, Breen RAM, Ormerod LP, Mittal R, Fisk M, Milburn HJ, Price NM, Bothamley GH, Lipman MCI (2016). Determinants of treatment-related paradoxical reactions during anti-tuberculosis therapy: a case control study. BMC Infect Dis.

[19] Chen C-H, Tsai J-J, Shih J-F, Perng R-P (1993). Tuberculous subcutaneous abscesses developing during chemotherapy for pulmonary tuberculosis. Scand J Infect Dis.

[20] Breen RAM, Smith CJ, Bettinson H, Dart S, Bannister B, Johnson MA, Lipman MCI (2004). Paradoxical reactions during tuberculosis treatment in patients with and without HIV co-infection. Thorax.

[21] Lai Z, Shi S, Fei J, Han G, Hu S (2018). A comparative study to evaluate the feasibility of preoperative percutaneous catheter drainage for the treatment of lumbar spinal tuberculosis with psoas abscess. J Orthop Surg Res.

[22] Ye F, Zhou Q, Feng D (2017). Comparison of the anteroposterior and posterior approaches for percutaneous catheter drainage of tuberculous psoas abscess. Medical science monitor: international medical journal of experimental and clinical research.

[23] Kagimoto A, Shibata S (2017). Resection of a Tuberculous abscess of the Thoracic Wall. Kyobu geka The Japanese journal of thoracic surgery.

