# Supplementary material for: Multiple subcutaneous tuberculous abscesses in a dermatomyositis patient without pulmonary tuberculosis: a case report and literature review
Source: BMC Infect Dis. 2020 Jun 12;20:409. doi: 10.1186/s12879-020-05137-w (PMC7291664; doi:10.1186/s12879-020-05137-w)
Supplement: Supplementary file 1 — Additional file 1 Table 1 Clinical characteristics of patients with tuberculous abscess in limbs. [file 12879_2020_5137_MOESM1_ESM.docx]

**Table 1 Clinical characteristics of patients with tuberculous abscess in limbs.**

| **Time** | **Age** | **Gender** | **Country** | **Abscess location** | **Underlying disease** | **TB history** | **active pulmonary TB** | **Treatment** | **Prognosis** | **References** |
| --- | --- | --- | --- | --- | --- | --- | --- | --- | --- | --- |
| 2019 | 49 | male | China | lower limbs and left forearm | No | Yes | Yes | HRZE | complete remission | [1] |
| 2019 | 35 | female | India | the left thigh and  the left upper limb | renal allograft recipient, diabetes | No | Yes | HRZE+quinolone drugs | complete remission | [2] |
| 2015 | 85 | male | Caucasian | the lateral side of the hip and thigh | rheumatoid arthritis | No | Yes | HREZ/HR | complete remission | [3] |
| 2013 | 45 | female | India | the right thigh | No | No | Yes | 2HREZ/4HR | complete remission | [4] |
| 2000 | 37 | female | Turkey | left lower half thigh, a popliteal fossa and a right tibial anterior surface | rheumatoid arthritis | Yes | Yes | HRZE+draining | complete remission | [5] |

Note: H, isoniazide; R, rifampin; Z, pyrazinamide; E, ethambutol.

1. Zeng Y, Liu Y, Xie Y, Liang J, Kuang J, Lu Z, Zhou Y: **Muscular Tuberculosis: A New Case and a Review of the Literature**. *Front Neurol* 2019, **10**:8.

2. Khandalvalli P, Nazneen S, Yadla M: **An unusual clinical presentation of tuberculous pyomyositis in a renal allograft recipient**. *Saudi Journal of Kidney Diseases and Transplantation* 2019, **30**(5):1175.

3. Migkos M, Somarakis G, Markatseli T, Matthaiou M, Kosta P, Voulgari P, Drosos A: **Tuberculous pyomyositis in a rheumatoid arthritis patient treated with anakinra**. *Clin Exp Rheumatol* 2015, **33**(5):734-736.

4. Modi M, Mate A, Nasta A, Gvalani A: **Primary tuberculous pyomyositis of quadriceps femoris in an immunocompetent individual**. *Case reports in infectious diseases* 2013, **2013**.

5. Mert A, Bilir M, Ozturk R, Tabak F, Ozaras R, Tahan V, Senturk H, Aktuglu Y: **Tuberculous subcutaneous abscesses developing during miliary tuberculosis therapy**. *Scandinavian journal of infectious diseases* 2000, **32**(1):37-40.
