# Supplementary figures and images for: Multiple subcutaneous tuberculous abscesses in a dermatomyositis patient without pulmonary tuberculosis: a case report and literature review
Source: BMC Infect Dis. 2020 Jun 12;20:409. doi: 10.1186/s12879-020-05137-w (PMC7291664; doi:10.1186/s12879-020-05137-w)

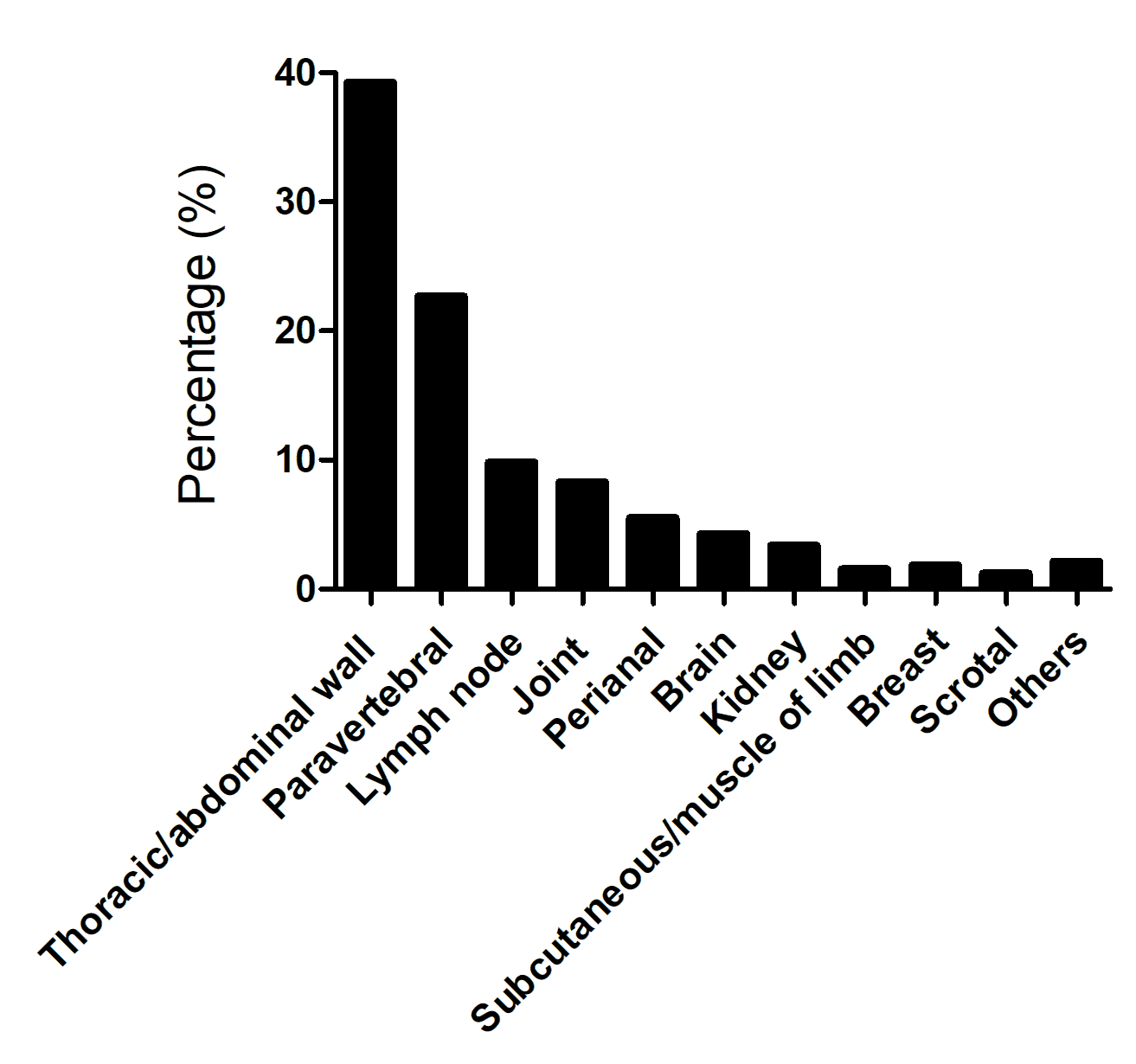

Supplement: Supplementary file 2 — Additional file 2. [file 12879_2020_5137_MOESM2_ESM.tif]
